# Brief therapy dog interactions may increase self-efficacy and lower perceived stress and cortisol in high school students: a preliminary study

**DOI:** 10.3389/fpsyg.2026.1876920

**Published:** 2026-08-11

**Authors:** Dawn K. Melzer, Deirdre M. Yeater, Madison DeRubis, Christiana Kohlroser, Barbara J. Pierce

**Affiliations:** 1 Department of Psychology, Sacred Heart University, Fairfield, CT, United States; 2 Department of Biology, Sacred Heart University, Fairfield, CT, United States

**Keywords:** animal-assisted intervention, canine, self-efficacy, stress, therapy

## Abstract

The present study examined changes in high schoolers’ perceived stress, general self-efficacy and cortisol levels after a brief interaction with a certified therapy dog. High school students completed self-report measures of perceived stress and general self-efficacy and provided salivary cortisol samples both before and after a 10-min interaction with a certified therapy dog. Following the interaction, students reported reductions in perceived stress scores, exhibited lower salivary cortisol concentrations and demonstrated increases in self-efficacy scores compared to before the interaction. Although higher self-efficacy scores were observed, general self-efficacy is generally regarded as a stable construct; therefore, these findings may reflect a temporary increase in perceived confidence or well-being rather than a lasting change in general self-efficacy. Overall, these findings should be interpreted as preliminary associations rather than evidence of a causal effect of the therapy dog interaction due to the single-group pre-post design and study limitations. Nevertheless, the results are consistent with previous research suggesting that brief therapy dog interactions may be associated with short-term improvements in psychological and physiological indicators of well-being. Additional controlled studies are needed to determine whether therapy dog interactions produce benefits beyond those attributable to other factors, such as social interaction or time away from academic demands.

## Introduction

In recent years, there has been a notable increase in stress levels among high schoolers, raising concerns about the well-being of this demographic (Stromájer et al., 2023). Preparation for exams, large assignments, peer competition, and the push to continue to higher education places a high amount of academic pressure on high school students. While manageable stress can motivate and enhance performance, chronic exposure to such stress can adversely affect adolescents’ mental and physical health, academic achievement, anxiety levels, and overall quality of life (Stromájer et al., 2023). Excessive academic stress can lead to lack of sleep, risks to physical health, increased substance abuse, and poor grades, as reviewed in Pascoe et al. (2020). When faced with a stressor, the body stimulates various processes aiming to preserve life and achieve evolutionary success. A crucial regulatory pathway in the maintenance of homeostatic processes is the hypothalamic–pituitary–adrenal axis which secretes cortisol, preparing the body for fight or flight. During short-term stress, cortisol levels rise, which can be seen as beneficial for promoting survival. However, long-term cortisol exposure can be detrimental, increasing the risk of developing mental disorders such as depression and anxiety (George et al., 2025).

Self-efficacy, defined as individuals’ beliefs in their ability to cope with challenges and achieve desired outcomes (Bandura, 1977), may also play a critical role in shaping stress management, and mental health across developmental stages. High levels of self-efficacy have been associated with better stress management, more confidence in use of coping skills, higher academic motivation, and greater emotional resilience (Bandura, 1977; Escalera-Chávez et al., 2018; Meng and Zhang, 2023). Research has found that low levels of self-efficacy are associated with increased anxiety and depression symptoms in adolescents (Fang et al., 2025; Kurnia et al., 2025).

Educators and school mental-health professionals have increasingly sought cost-effective strategies to support adolescents’ well-being, with animal-assisted interventions (AAI) emerging as a promising option (Brelsford et al., 2017; Moe et al., 2025; Parbery-Clark et al., 2021). These programs typically involve interactions with animals, most commonly dogs, scheduled during periods of heightened academic stress such as examinations (Peel et al., 2023). Research suggests that such interactions may reduce stress by lowering blood pressure and heart rate, improving mood and perceived self-efficacy, increasing oxytocin release, enhancing emotional regulation, decreasing self-perceived stress and fostering social connectedness (Banks et al., 2008; Banks et al., 2018; Jimenez et al., 2023; Nagasawa et al., 2015; Napier et al., 2021; Peel et al., 2023). Empirical studies consistently demonstrate reductions in psychological stress and cortisol following interactions with dogs, including among college students after 60-min sessions with non-therapy dogs (Jimenez et al., 2023) and after 10-min sessions with shelter animals (Pendry and Vandagriff, 2019). Interactions with trained therapy dogs may have additional calming effects due to their specialized certification process, as evidenced by cortisol reductions in children after structured exposure sessions (Meints et al., 2022) and in healthcare workers following 45-min interactions (Barker et al., 2005). Evidence suggests AAI may also enhance self-efficacy, as demonstrated in adult psychiatric patients following a 12-week program involving various therapy animals (Berget et al., 2008). Despite growing adoption of AAI programs in high schools, research on this population remains limited due to restricted access to this population and demographic data collection.

In the current study, high school students’ stress and self-efficacy levels were assessed using the Perceived Stress Scale (PSS) and the General Self-Efficacy Scale (GSES) before and after interacting with a certified therapy dog for 10-min in small peer groups (Cohen et al., 1983; Schwarzer and Jerusalem, 1995). This time was selected to more closely resemble the real-time interactions that occur between therapy dogs and students when the dogs are visiting, which usually occurs in-between classes or during short breaks. The group dynamic was included to additionally replicate the scenario that occurs during the animal assisted intervention programs at schools. To explore the physiological stress response, salivary cortisol levels were collected before and after the interaction in addition to responding to the questionnaires. It was hypothesized that a 10-min interaction with a certified therapy dog would be associated with lower perceived stress and salivary cortisol levels and higher self-efficacy scores following the interaction compared to baseline.

## Methods

### Participants

Seventy-five high school students from two different northeast high schools participated in the current study. One high school was a magnet school (*N* = 60) and the other was a public school (*N* = 15). Although a formal *a priori* power analysis was not conducted, the sample size was comparable to or larger than those used in previous studies (Banks et al., 2018; Grajfoner et al., 2017; Jimenez et al., 2023). The students ranged in age from 14 to 19 (M = 16.3 yrs., SD = 0.99). In collaboration with the primary investigators, science teachers from both high schools facilitated student recruitment. All procedures involving humans in this study were approved by the Sacred Heart University Institutional Review Board (IRB Protocol #230515B). Given the age of the students, a parental consent form was sent home to parents and after signing and returned, students completed an Institutional Review Board (IRB) Assent form before participating in the study. Due to internal high school IRB guidelines at one of the high schools, the only demographic information collected was age. To be consistent, no further demographic information was collected at the second high school. The students were not given any incentives to participate.

### Materials and procedure

The research protocol was the same at both high schools and all samples were collected between 10 a.m. and 12 p.m. The participants entered a classroom with desks and chairs. They were greeted by the research assistants, and the participants were asked if they had abstained from eating or drinking for the previous 60 min to ensure accuracy of saliva-cortisol testing. If they had complied with the pre-testing instructions, they were given the Youth Assent form to read and sign. If participants had not complied, they were asked to return 60 min later, if still within the testing window, if they were able to abstain from eating or drinking during that time (*N* = 2) or were dismissed from the study (*N* = 1) which left us with seventy-four participants in total. The research assistants then verbally informed the participants about the experiment activities and offered to answer any questions the participants had regarding the study. Participants were also asked if they had any canine allergies or fear of dogs. If they answered in the affirmative, their response was recorded on their consent form. After confirmation that the participant was willing and able to participate, they were assigned a participant number and were informed they would be using that number when completing the surveys and cortisol tests during the study.

#### Therapy dogs

A total of five certified therapy dogs participated in the study across the two schools. The dogs varied in size (20–60 lbs), age, and breed. All dogs met the criteria for certification set by the appropriate therapy dog national organizations. To ensure the safety and well-being of the dogs involved in the study, all procedures were approved by the Sacred Heart University Institutional Animal Care and Use Committee (IACUC Protocol # 2019-R004).

#### Pretests

A timer was set for 15 min after the participants completed the Youth Assent forms to ensure baseline cortisol levels reflected participant levels when they first entered the study space. Salivary cortisol concentrations peak within 15–20 min after exposure to a stimulus (Barker et al., 2005; Lightman, 2008; Russell and Lightman, 2019). During this 15-min window, the participant was given a QR code for either the PSS or the GSES and was asked to complete the questionnaire on their phone or tablet. The administration of these surveys was counterbalanced. Once the first survey was completed, the participants scanned the second QR code and completed the second survey.

##### The Perceived Stress Scale (PSS)

The PSS is a ten-item scale that measures the degree an individual perceives a scenario as stressful in the past month. Questions assess current stress levels and emotional states. For example, “In the last month, how often have you been upset because of something that happened unexpectedly?” A five-point response scale is used to answer each question ranging from “never” to “very often.” Scores range from 0 to 40, with higher scores indicating higher levels of perceived stress. Although the PSS is designed to assess perceived stress experienced over the previous month, it was administered both before and after the therapy dog interaction to examine whether participants’ perceptions of stress changed following the intervention, consistent with previous studies employing a similar pre-post design (Beasley et al., 2022; Ward-Griffin et al., 2018).

##### The General Self-Efficacy Scale (GSES)

The GSES is a ten-item scale that measures the self-efficacy one has based on experiences from the past and the tendency of the individual to credit success to skill versus luck. Statements such as “I give up easily” or “I am a self-reliant person” are given to the respondent. A five-point response scale is used to answer each statement ranging from “strongly disagree” to “strongly agree.” GSES scores range from 10 to 40, with higher scores indicating higher levels of self-efficacy.

##### Cortisol measurements

Saliva samples from participants were collected using the Salimetrics® Salivabio Oral Swab (SOS) passive drool method 15 min after arrival at the study (baseline). Participants were given a vial/receptacle and a sealed cotton swab. They were instructed to open the wrapper containing the cotton swap without letting the swab touch their fingers. They placed the swap directly under their tongues and timed for 90 s. After 90 s, the participants opened the vial, pushed the saliva filled cotton swab into the vial, without touching it with their fingers, and closed the vial lid. The samples were labeled with participant’s number and were immediately placed on ice (below 4 °C) until transferred to the laboratory. Samples were stored at −80 °C until processing.

#### 10-min therapy dog interaction

After completion of the pretests, participants were brought into a separate classroom where two to three certified therapy dogs were in segregated areas of the classroom space. Four to five participants were assigned to sit with each dog for 10 min. A rug and cushions were available for the dog and the participants on the floor. Most students wished to sit on the floor with the dog, though chairs were available if the student was uncomfortable or unable to sit on the floor for the entire 10-min period. Each therapy dog’s human handler was present during the entire session. Participants were informed they could interact with the dog as little or as much as they were comfortable. Interactions included petting the dog, speaking to the dog, speaking with the handler, talking to other participants in the group, using commands with the dog (e.g., asking the dog to sit or roll over), giving the dog a treat or just sitting near the dog.

#### Posttests

After interaction with the therapy dog, participants were taken back into the original classroom space to complete the same three assessments they had completed prior to the therapy dog interaction (e.g., PSS, GSES, cortisol). During the fifteen-minute period following the interaction with the therapy dog, participants were once again presented with the QR code for the PSS or GSES, which were counterbalanced, and responded to each survey on their phone or device. After participants answered each survey, they waited for the 15-min time to elapse and were once again administered the Salimetrics® Salivabio Oral Swab (SOS) passive drool protocol as outlined above. After completing the second cortisol collection, participants were debriefed and thanked for their participation.

##### Cortisol assays

We used Salimetrics® Assay #1–3,002 Salivary Cortisol Elisa Kit for all salivary cortisol determinations. We followed all general procedures set forth in the Salimetrics® assay protocol Rev. 04.19.19 (1–3,002). Saliva samples were thawed and centrifuged at 1,500 ×*g* for 15 min. Samples were transferred from the collection tubes to Eppendorf tubes for ease of use and re-freezing after aliquot was taken. All samples were processed at room temperature. Assay kits were removed from the refrigerator and allowed to acclimate to room temperature for 90 min prior to use. Each kit contains a 96-well microtitre plate which was kept in the foil pouch until use to avoid humidity on the plate. Unlike the kit procedure, we measured all standards and saliva samples in triplicate in an effort to decrease the need to re-measure samples. Thus, we were able to measure the saliva of 11 subjects pretest and posttest per plate. We used a BMG Labtech SPECTROstar Omega Microplate Reader, and all samples were read at a discrete wavelength of 450 nanometers.

### Data analysis

#### Perceived Stress Scale (PSS) and General Self-Efficacy Scale (GSES)

A paired two-sample t-test was used to compare participants’ pretest and post-test scores on the PSS and GSES using IBM SPSS Software Version 29.0.1.0. Seven participants’ scores were excluded from GSES analyses because they did not successfully complete both the GSES pre and posttests. Therefore, sixty-seven participants’ data were included in the GSES analyses. The GSES and PSS were counterbalanced in how they were administered, both accessed through a QR code, and there were no internet issues during either research session. We attribute the issue to a failure to confirm survey completion and in future studies participants will be required to display a submission confirmation to the researcher. To account for the possibility that items contributed differently to the constructs, McDonald’s omega (*ω*) was used to test reliability of these measures. No correction for multiple comparisons was applied as the paired-samples *t* tests evaluated distinct data with preplanned hypotheses.

#### Cortisol measurements

Absorbance values of saliva samples obtained from the microplate reader were input into a four-parameter logistic curve using the on-line data analysis tool (MyAssays® Ltd., 2012) to determine salivary cortisol concentrations. Sample replicates which had a %CV in cortisol concentration greater than 15% from the %CV of the other two replicates was considered an outlier and was removed from the analysis. No samples were re-measured. We considered samples as invalid if the levels of cortisol exceeded the calibrated range of standards (*n* = 2) and those samples were removed from the analysis. The average coefficient of variation for intra-assay was 10.2%, SE 0.76, and inter-assay was 11.1%, SE 1.3. A paired-sample t-test was conducted using IBM SPSS Software Version 29.0.1.0 to compare the salivary cortisol concentration (mcg/dl) of students prior to the interaction with a therapy dog (pretest) and after the interaction with a therapy dog (posttest).

## Results

### Perceived Stress Scale (PSS)

**Figure 1:**
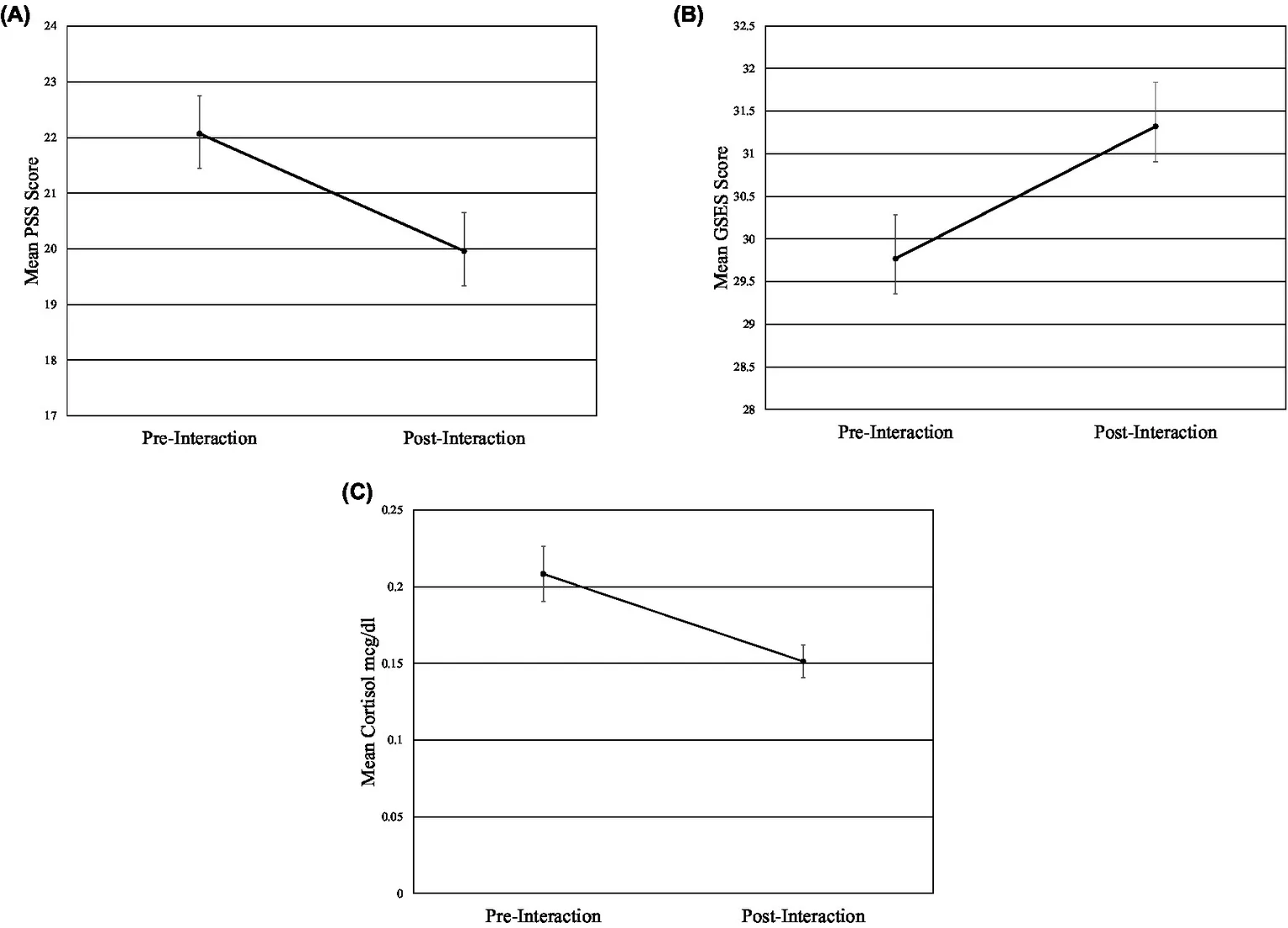
**(A)** Total mean score on Perceived Stress Scale (PSS) survey achieved by high school students before and after a 10-min interaction with a therapy dog (*n* = 74). Error bars indicate standard error. Scores were significantly different at *t*(73) = 2.29, *p* = 0.025. **(B)** Total mean score on General Self-Efficacy Scale (GSES) survey achieved by high school students before and after a 10-min interaction with a therapy dog (*n* = 66). Error bars indicate standard error. Scores were significantly different at *t*(66) = 2.27, *p* = 0.027. **(C)** Mean cortisol concentration (mcg/dl) measured in the saliva of high school students before and after a 10-min interaction with a therapy dog (*n* = 73). Error bars indicate standard error. Concentrations were significantly different at *t*(72) = 4.69, *p* < 0.001.

To assess internal consistency, separate reliability analyses were conducted for the two subscales of the PSS. The scale demonstrated good internal consistency at pre-test for perceived helplessness (*⍵* = 0.82) and satisfactory consistency for lack of self-efficacy (*⍵* = 0.72). For the PSS post-test the scale demonstrated good internal consistency at pre-test for perceived helplessness (*⍵* = 0.84) and satisfactory consistency for lack of self-efficacy (*⍵* = 0.75). Participants scored significantly lower on the PSS posttest after the 10-min therapy dog interaction (*N* = 74, *M* = 19.96, *SD* = 5.4) than the pretest (*N* = 74, *M* = 22.07, *SD* = 5.9), 2.29, *p* = 0.025, *d* = 0.27, 95% CI [0.03, 0.49]; Figure 1A. On average, PSS scores decreased by 2.12 points after the interaction. There were no significant effects of age on the mean PSS scores or changes in scores from the pre- to post-test scores.

### General Self-Efficacy Scale (GSES)

The GSES demonstrated good internal consistency at pre-test (*⍵* = 0.83) and post-test (*⍵* = 0.84). Participants scored significantly higher on the GSES posttest (*N* = 67, *M* = 31.12, *SD* = 3.89) after the 10-min interaction with the therapy dog than on the GSES pretest (*N* = 67, *M* = 29.58, *SD* = 4.17), *t*_(66)_ = 2.27, *p* = 0.027, *d* = 0.28, 95% CI [0.52, 0.03]; Figure 1B. On average, GSES scores increased by 1.54 points after the intervention. There were no significant effects of age on the mean GSES scores or changes in scores from the pre- to post-test scores.

### Cortisol measurements

The mean cortisol concentration measured in the saliva of participants after (*N* = 73, *M* = 0.148 mcg/dl, *SD* = 0.09) the 10-min interaction with the therapy dog was significantly lower than before the interaction (*N* = 73, *M* = 0.204 mcg/dl, *SD* = 0.15), t_(72)_ = 4.69, *p* < 0.001, *d* = 0.55, 95% CI [0.032, 0.08]; Figure 1C. On average, salivary cortisol concentration of participants decreased by 0.056 mcg/dl after the interaction.

## Discussion

The present study investigated whether a brief, 10-min interaction with a certified therapy dog was associated with changes in perceived stress, salivary cortisol, and general self-efficacy among high school students. Following the interaction, participants demonstrated lower perceived stress scores, lower salivary cortisol concentrations, and higher general self-efficacy scores compared with their pre-intervention assessments. However, because the present study employed a single-group pre-post design, due to the constraints of working with minors in a high school setting, these findings should be interpreted as preliminary associations rather than direct evidence that the therapy dog interaction directly caused the observed changes.

The present findings are consistent with previous research that have shown that college students exposed to therapy dog interactions had significant reductions in levels of state anxiety, perceived stress, and displayed improvement in their well-being (Grajfoner et al., 2017, Banks et al., 2018). Extending this literature to a high school population is noteworthy, as few studies have examined therapy dog interventions with adolescents. Although additional controlled research is needed, the present findings suggest that brief therapy dog interactions may be associated with short-term changes in multiple indicators of psychological well-being in this understudied population.

To our knowledge, ours is the only study to observe an increase in self-efficacy among high school students following a 10-min interaction with a therapy dog. While this finding aligns with previous work suggesting that interactions with dogs may increase self-efficacy in their owners (Napier et al., 2021), it should be interpreted with some caution. General self-efficacy is considered a relatively stable construct during adolescence, developing through repeated mastery experiences, observational learning, and supportive social environments (Kleppang et al., 2023). However, longitudinal research suggests that self-efficacy may show some variability during adolescence (Grevenstein and Bluemke, 2017; Wells et al., 2018). Even so, the increase observed after the brief interaction may reflect a temporary enhancement in participants’ confidence immediately following a positive experience rather than a lasting change in general self-efficacy.

Several studies have similarly examined the short-term changes following therapy dog and non-therapy dog interactions on college student cortisol levels using a pre-post sampling design (Delgado et al., 2018; Jimenez et al., 2023; Krause-Parello et al., 2012). This design is commonly used to examine relative changes in salivary cortisol due to its ease of deployment in situations where multiple baselines/repeated measures with participants are not feasible. Similar to previous pre-post studies, saliva samples in the present investigation were collected within a standardized time window to reduce, although not eliminate, the influence of circadian variation (Barker et al., 2010: Kelker et al., 2025: Krause-Parello et al., 2012). Krause-Parello et al. (2012) collected saliva samples before and after a 20-min hands-on canine interaction between the same 2 h every morning. Kelker et al. (2025) measured salivary cortisol of pediatric emergency room patients between 10 a.m. and 4 p.m. before and after a 10-min canine interaction. Consistent with our findings, all studies reported significant reductions in salivary cortisol levels in participants following a canine interaction, though the absence of a control group in the current study prevents us from distinguishing between intervention effects and circadian variation. Although cortisol levels are known to be highly variable between individuals, and are influenced by circadian and ultradian rhythms, stressful situations and/or stress reduction activities can influence circulating cortisol levels (Lightman, 2008: Russell and Lightman, 2019). More recently, studies have shown that relative, short-term changes in salivary cortisol levels can act as an appropriate physiological measure of acute changes in stress levels of subjects (see Smyth et al., 2013 for review).

Our findings contribute to this growing body of literature by demonstrating initial evidence for the short-term changes in a high school population after a therapy dog interaction, which has received less empirical attention than other populations. Although the observed reductions in perceived stress and cortisol are encouraging and consistent with prior work, alternative explanations, including the passage of time, participation in a pleasant activity, or social interaction with peers and adults, cannot be ruled out due to our inability to conduct a control group, thus there is room for future research on this population.

### Limitations and future directions

Research on therapy dog intervention effectiveness in the high school population is still in its infancy. Questions persist about whether therapy dogs provide unique benefits to stress and self-efficacy compared to other interventions or interactions with non-therapy dogs (e.g., non-animal therapeutic intervention, robotic dog, other animals, or drawing) (Banks et al., 2008; Gardiánová and Hejrová, 2015). Future research should employ randomized controlled designs comparing therapy dog interactions with appropriate control or comparison conditions to determine whether therapy dogs provide benefits beyond those associated with social interaction, participating in a calm activity, or resting. A longitudinal study examining the long-term effects of brief therapy dog interactions, or multiple interactions, on additional outcomes (e.g., academic performance, academic self-efficacy, and anxiety) would provide valuable insight into their sustained impact.

Given the inherent variability of salivary cortisol measures, it may be beneficial to collect multiple baseline samples, consistent with prior research on intraindividual variability (Danese et al., 2024; Gandenberger et al., 2022). In addition, measurement of oxytocin levels in conjunction with cortisol levels will provide support for the mechanistic explanation that an increase in oxytocin contributes to a decrease in cortisol levels (Jimenez et al., 2023; Stauch, 2024).

Due to school policies, we were unable to collect demographic information on the high school sample and there was an issue collecting post-intervention self-efficacy data that reduced our sample size for that variable. The lack of demographic data limits our ability to determine whether the sample was representative of the broader high school population and prevents subgroup analyses that could identify whether the observed associations differed by gender, clinical diagnoses, socioeconomic status, culture, or age. Future studies should prioritize the collection of demographic information and recruit samples across multiple schools to improve generalizability and allow for further analyses. In addition, standardized procedures for verifying questionnaire completion immediately after administration should be implemented to minimize missing data.

As mentioned, the PSS assesses individuals’ perceptions of stress experienced over the preceding month, rather than acute changes in stress. Though previous studies have successfully employed the PSS using a similar pre-post design to that used in the current study (Beasley et al., 2022; Ward-Griffin et al., 2018), the measure may not be optimal in detecting fluctuations in stress over a 10-min period. Consequently, the observed changes should be interpreted with appropriate caution and may reflect temporary changes in participants’ perceptions of stress rather than broader changes in perceived stress over the previous month. Future research could strengthen the assessment of immediate intervention effects by incorporating measures specifically designed to capture acute stress or state anxiety.

## Conclusion

Although preliminary, this study extends the growing body of research examining the potential role of therapy dog interventions in supporting adolescent well-being by focusing on high school students and incorporating both psychological and physiological measures. Although the single-group pre-post design precludes causal conclusions, the findings provide preliminary evidence that brief interactions with certified therapy dogs were associated with lower perceived stress, reduced salivary cortisol concentrations, and greater self-reported confidence in managing challenges and achieving desired goals immediately following the interaction. Future studies employing randomized controlled designs, collection of demographic data, and additional comparison conditions are needed to determine whether these associations are uniquely attributable to therapy dog interactions. Until such evidence is available, these findings support continued investigation of therapy dog programs as a potentially promising component of comprehensive, low-cost, school-based wellness initiatives for adolescents.

## Data Availability

The raw data supporting the conclusions of this article will be made available by the authors, without undue reservation.
